# Controlling Hybrid Polyhydroxyurethane Adhesive and Rheological Properties by Partial Carbonation of Biobased Epoxy Monomer

**DOI:** 10.1002/marc.202400542

**Published:** 2024-07-29

**Authors:** Pierre Delliere, Dorian Laborie, Sylvain Caillol, Camille Bakkali‐Hassani

**Affiliations:** ^1^ ICGM, Univ Montpellier, CNRS, ENSCM Montpellier 34293 France

**Keywords:** adhesive, biobased PHU, partial carbonation, vitrimer

## Abstract

Controlling hybrid material properties by simple monomer design offers an elegant pathway to prepare thermoset adhesives with tunable properties. Herein, biobased hybrid polyhydroxyurethane/polyepoxy is prepared starting from partially carbonated cashew nut shell epoxy derivatives (NC514) and *m*‐xylene diamine (MXDA). The curing reactions, that is, epoxy‐amine and cyclic carbonate aminolysis, monitored by ATR‐IR spectroscopy at 50 °C are found to be concomitant yielding highly homogeneous materials. Hybrid networks are extensively characterized by swelling tests, TGA, DMA, DSC, tensile tests, rheology, and lap‐shear‐test on aluminum substrates. The introduction of hydroxyurethane moieties within the epoxy‐amine networks enhanced the adhesion properties (up to 20% compare to neat epoxy resins) by combining hydrogen bonding capability and vitrimeric properties (thermoset able to flow). Rheological characterizations and reprocessing tests demonstrated that hybrid adhesives with up to 47 mol% of cyclic carbonate groups are capable of covalent exchange (internally catalyzed by tertiary amine) while keeping similar thermomechanical properties and enhanced adhesion strength compare to the permanent epoxy network.

## Graphical abstract

Partial carbonation of epoxy monomers offers a simple pathway to tune the viscoelastic, adhesive, and thermomechanical properties of the resulting hybrid materials when reacted with primary amines. Fully biobased structures and partially recyclable/reprocessable thermosets (vitrimer‐like) can be thus obtained in a catalyst‐free approach.

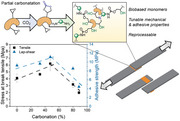

## 1 Introduction

Adhesives have a prominent role in numerous applications where an efficient bonding between objects is necessary.^[^ ^1^, ^2^ ^]^ Adhesive's bonding can be traced back to ancient times from early birch bark pitches and bitumen glues, there followed materials made from animal collagen or coagulation of egg albumin. The development of synthetic polymers during the last century has offered a wide range of adhesive formulations with tunable thermomechanical properties, setting times or processes.^[^ ^3^ ^]^ When high performances are targeted, most adhesives rely on crosslinking (covalent networks or thermosets) to achieve outstanding adhesion properties. Among adhesives, epoxies^[^ ^4^, ^5^ ^]^ and polyurethanes^[^ ^6^, ^7^ ^]^ (PUs) stand as important polymeric adhesives able to bond material such as wood, metals, rubber, and ceramics. Epoxy thermosets possess high strength, chemical and thermal resistance, good durability and relatively low curing temperatures when primary amines are employed as hardener. Alternatively, PU adhesives are generally flexible at low temperatures, resistant to fatigue, impact, and exhibit good durability.^[^ ^8^ ^]^

Merging epoxy and urethane chemistries introduces fresh avenues for exploring original materials with distinctive or even enhanced capabilities.^[^ ^8^, ^9^ ^]^ Nevertheless, a major drawback in PU systems is the use of highly toxic aromatic isocyanates such as methylene diisocyanate or toluene diisocyanate.^[^ ^11^ ^]^ Polyhydroxyurethane (PHU) and derivatives have been extensively studied to replace PU produced from isocyanate monomers in the so‐called non‐isocyanate polyurethane (NIPU) strategy.^[^ ^12^, ^13^, ^14^, ^15^ ^]^ Hydroxy‐urethane moieties are classically obtained from aminolysis of cyclic carbonates (5 to 8‐membered heterocycles) with primary amines.^[^ ^16^, ^17^, ^18^ ^]^ To produce PHU, five‐membered cyclic carbonates (5CCs) have been the most employed monomers as it can be generated from the corresponding epoxides, carbon dioxide, and suitable catalysts.^[^ ^19^ ^]^ This approach also exhibits some limitations related to the low reactivity of bis‐5‐CC toward aminolysis,^[^ ^20^ ^]^ the reversibility of carbonate aminolysis, the occurrence of side reactions^[^ ^21^ ^]^ (mainly urea formation), and the formation of a dense hydrogen bond networks (high viscosity).^[^ ^22^ ^]^ Furthermore, epoxy‐PHU hybrids have been investigated to produce adhesives^[^ ^23^, ^24^ ^]^ but also foams^[^ ^25^ ^]^ at room temperature employing judiciously the exothermic ring‐opening of epoxides thus reducing the gelation time. This approach allows to overcome the limitation of PHU chemistry, especially the low reactivity of 5CCs toward aminolysis,^[^ ^26^ ^]^ but also improves the thermo‐mechanical and adhesive properties of neat epoxides even if this behavior has not been rationalized yet. Epoxy‐PHU hybrids can be obtained via two different routes either by (i) reacting fully carbonated monomer and neat epoxy monomers with primary amines ^[^ ^9^ ^]^ or by (ii) partial carbonation of epoxy monomers and the subsequent crosslinking reaction with primary amine.^[^ ^27^ ^]^ Partial carbonation of epoxy monomers appears as an elegant strategy to design resins with tunable epoxy/carbonate contents. In addition, the introduction of exchangeable covalent bonds ^[^ ^28^, ^29^, ^30^ ^]^ (hydroxyurethane moieties) and internal tertiary amine ^[^ ^31^, ^32^ ^]^ (from the epoxy ring‐opening by primary amine) could also bring interesting properties such as reprocessability, recyclability or debonding properties.^[^ ^33^, ^34^ ^]^

Herein, we explored the partial carbonation of a commercially available biobased epoxy derived from cashew nutshell (NC514) to prepare fully biobased adhesives with tunable properties. The kinetic of the carbonation reaction (CO_2_ fixation) as well as the crosslinking reaction with a biobased diamine (*m*‐xylene diamine) was studied for all formulations by FTIR analysis. All materials were extensively characterized by combining gel content determination, thermal and thermo‐mechanical analysis (DSC, TGA, DMA, rheology, and tensile test). The impact of partial carbonation was evaluated for both adhesion on aluminum substrates and dynamic properties (covalent exchange) by performing lap‐shear tests, and rheological characterization (stress relaxation experiments and reprocessing cycles).

## 2 Experimental Section

### 2.1 Materials

NC514 epoxy resin was supplied by Cardolite, tetrabutylammonium bromide (TBAB, 99%) by TCI Chemicals, Carbon dioxide (CO_2_, 99.995%) by Linde, *m*‐xylene diamine (MXDA, 99%) and 1,3,5‐trioxane (99.9%) were acquired from Sigma–Aldrich, ethyl acetate (99%) by Fisher Scientific and CDCL_3_ (99.5%) by Eurisotop. Dimethyl formamide (DMF, 99.5%). Aluminum alloy 5005A plates (AlMgI) were supplied by Thessenkrupp. All chemicals were used as received.

### 2.2 Carbonation Procedure

In a typical synthesis, 70.0 g of NC514, 3.5 g of tetrabutylammonium bromide (5 wt.%), and 140 mL of ethyl acetate were added to a 300 mL autoclave. The autoclave was heated to 80 °C and then pressurized with 20 bars of CO_2_ from 15 to 240 min depending on the extent of carbonation desired. For partial carbonation, the reaction was quenched by dipping the reactor in ice‐cold water and rapidly degassing while maintaining mechanical agitation until both temperature and pressure stabilized to ambient conditions. After depressurization, tetrabutylammonium bromide was removed from the crude mixture by liquid‐liquid extraction with de‐ionized water (three times). The organic layer was then dried over MgSO_4_. Ethyl acetate was then evaporated at 70 °C under high vacuum (10^−3^ mbar).

**Figure 1:**
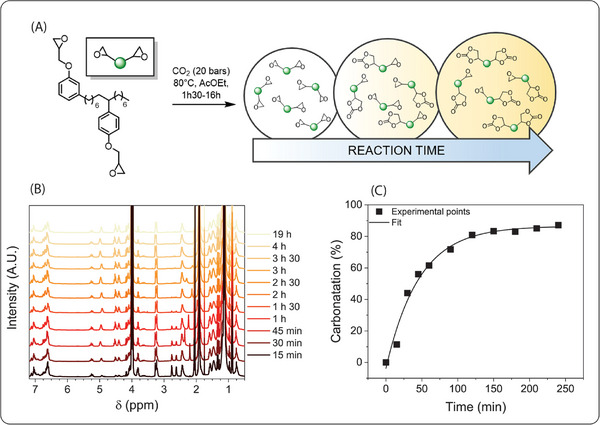
A) Partial carbonation of NC514 (B) and (C) ^1^H NMR kinetic experiments of carbonation with [NC514]_0_ = 0.5 g mL^−1^ and tetrabutylammonium bromide (TBAB, 5 wt.%) under 20 bar of carbon dioxide at 80 °C in ethyl acetate.

A kinetic study was conducted by taking aliquots from the reaction medium followed by ^1^H NMR titration of the carbonate content (spectra in **Figure** **1**B). The evolution of epoxy conversion as a function of reaction time is available in Figure 1C. This kinetic plot was employed to target an array of partially carbonated NC514 resins. Table S1 (Supporting Information) reports their respective carbonation time, epoxy content, and carbonate content.

### 2.3 Characterization of Resins

#### 2.3.1 Nuclear Magnetic Resonance (NMR)

All NMR experiments were carried out at 298 K on a BRUKER Avance III spectrometer.

The carbonation kinetic was established using Equation (1) and an unreactive methyl peak (0.7 ppm) as the internal standard. In Equation (1), *A*_*Epox* *NC*514_ and *A*_*Methyl* *NC*514_ stand for the area of the epoxy (2.55 – 2.80 CH_2_, ppm) and methyl (CH_3_, 0.7 ppm) peaks in the uncarbonated NC514. *A_Epoxy_* corresponds to the area of the partialy carbonated resins’ epoxy peak (2.55 – 2.80 CH_2_, ppm).

(1) $$Epoxy\;conversion\;\left(a\right)\;=\frac{\frac{A_{Epoxy\;NC514}}{A_{Methyl\;NC514}}-\frac{A_{Epoxy}}{A_{methyl}}}{\frac{A_{Epoxy\;NC514}}{A_{Methyl\;NC514}}}\;$$

To determine the epoxy and carbonate content of each resin, known masses of resin and an internal standard (1,3,5‐trioxane) were introduced into and NMR tube and dissolved with 600 µL of CDCL_3_. The epoxy and carbonate content were calculated using Equations (2) and (3). In these equations, *A_epoxy_* stands for the area of the CH_2_ peak of epoxy functions (2.55 – 2.80 ppm), *A_Carbonate_* for the area of the CH of cyclocarbonate functions (4.60 – 4.40 ppm) and *A_Trioxane_* for the area of the internal standard's CH_2_ groups (5.16 ppm). *M_Trioxane_* and *m_Trioxane_* are respectively the molecular weight and the mass of 1,3,5‐trioxane introduced in the NMR tube. *m_Resin_* stands for the mass of epoxy or epoxy‐carbonate resin poured in the NMR tube. Finally, *nH_Trioxane_*, *nH_Epoxy_* mass and *nH_Carbonate_* correspond to the number of hydrogens under each peak, that is, 6, 2, and 1 hydrogen atoms.

(2) $$\begin{array}{ccc} & & Epoxy\;content\;\;\left(mmol.g^{-1}\right)=\\ & & \frac{A_{epoxy}\;\times \;nH_{Trioxane\;}\times \;m_{Trioxane}}{A_{Trioxane\;}\times \;nH_{Epoxy}\;\times \;m_{Resin}\;\times \;M_{Trioxane}}\; \end{array}$$

(3) $$\begin{array}{ccc} & & Carbonate\;content\;\;\left(mmol.g^{-1}\right)=\\ & & \frac{A_{Carbonate\;}\times \;nH_{Trioxane}\;\times \;m_{Trioxane}}{A_{Trioxane}\;\times \;nH_{Carbonate}\;\times \;m_{Resin}\;\times \;M_{Trioxane}}\; \end{array}$$

#### 2.3.2 Fourier‐Transformed Infrared Spectroscopy (FTIR)

A Thermo‐scientific Nicolet iS50 equipped with a Specac Golden Gate attenuated total reflection (ATR) heating cell was employed to acquire all FTIR spectra.

The characterization of the resins and materials were performed at 25 °C using a standard deuterated L‐alanine doped triglycerine sulfate (DTGS) detector with an accumulation of 32 scans for both spectra and background (air). FTIR spectra of epoxy and epoxy‐carbonate resins are available in Figure S1A (Supporting Information). FTIR spectra of epoxy and epoxy‐PHU hybrid materials are available in Figure S1B (Supporting Information).

The curing kinetic of epoxy and epoxy‐PHU hybrids was assessed in isothermal mode at 50 °C for 100 min. A liquid nitrogen‐cooled mercury cadmium telluride (MCT/A) detector was employed to allow the collection of high‐quality spectra each 30 s using only 8 scans (5 s acquisition time).

The epoxy and carbonate conversions (α) were assessed using Equation (4). In this Equation, *A*_0_ corresponds to the area of the carbonate peak (1750–1860 cm^−1^) or the epoxy peak (890‐940 cm^−1^) of the first acquired spectra. *A*_0_ is slightly underestimated due to the mixing time required to reach homogeneous mixtures (2 min at 25 °C). *A_t_* is the area of the carbonate or epoxy peak at a given curing time.

(4) $$FTIR\;conversion\;=\;\frac{A_{0}-A_{t}}{A_{0}}\times 100$$

### 2.4 Preparation and Reprocessing of Hybrid PHUs

Epoxy‐PHU hybrids were prepared by combining partially carbonated resins with MXDA. In a typical procedure, a partially carbonated resin was preheated to 50 °C in a 50 mL polypropylene container. After the addition of MXDA, the components were stirred at 2500 RPM for 4 min with planetary mixer (SpeedMixer DAC 400.2 VAC‐P from Hauschild). Once homogenized, the mixture was poured into PTFE molds and cured at 80 °C for 24 h. The hybrid materials were named PHU‐X, X being the carbonation percentage of the initial resin. The masses of NC514, partially carbonated NC514, and MXDA for each PHU hybrid are available in Table S2 (Supporting Information). Fully carbonated NC514 resin was excluded from the study as three days are required to manufacture it. Furthermore, the polymerization of a fully carbonated NC514 with MXDA leads to a thermoplastic material consequently falling out of the scope of the present study.

As detailed earlier, epoxy‐PHU hybrids display a dual reactivity. Equation 5 was employed to determine the mass of MXDA (*m_MXDA_*) for each formulation. In Equation (5), *m_resin_* stands for the mass of partially carbonated or NC514 resin. The epoxy and carbonate content emerge from Equations (2) and (3), respectively. Finally, the NH and NH_2_ content were obtained from Equation (6).

(5) $$m_{MXDA}=m_{Resin}\;\times \left(\frac{Epoxy\;content}{NH\;content}+\frac{Carbonate\;content}{NH_{2}\;content}\right)$$

(6) $$M_{MXDA}=\frac{4}{NH\;content\;}\;=\frac{2}{NH_{2}\;content}\;$$

Finally, a short reprocessability study was carried out on the most promising sample (PHU‐47). To do so, ≈10 g of samples were cut into rough polygon pieces (≈30 – 40 mm^3^). The pieces were then placed in a stainless‐steel mold (100 × 50 mm) sandwiched between PTFE‐protected stainless‐steel plates (200 × 200 mm) and pressed at 150 °C under three tons for 6 h. The time and temperature conditions were based on stress‐relaxation experiments.

### 2.5 Characterization of PHUs

#### 2.5.1 Tensile and Lap‐Shear

Tensile tests were performed at room temperature (22 ± 1 °C) on seven dumbbell‐cut dog‐bone specimens (ISO 527–2 5B). Tensile tests were performed on an Instron 5900 apparatus at a deformation rate of 5 mm min^−1^. Stress‐strain curves were recorded and then processed using OriginPro 2021.

Lap‐shear tests were conducted using 100 × 25 × 2 mm Aluminum alloy 5005A plates with an adhesion area of 25 × 10mm^2^. The deformation rate was set at 10 mm min^−1^. The adhesive strength was determined using Equation (7).

(7) $$Adhesive\;strength\;=\;\frac{Maximum\;force}{Adhesive\;area}$$

#### 2.5.2 Differential Scanning Calorimetry (DSC)

DSC measurements were conducted on a DSC200F3 from NETZCH. 8–12 mg of sample were placed in 40 µl aluminum crucibles with pierced lids and heated from −50 to 200 °C at 10 °C min^−1^ under a nitrogen flow of 20 mL min^−1^. In all cases, no residual curing enthalpy could be observed. T_g_ (DSC) was taken as the mid‐point of the specific heat capacity drop. Data were exploited using Proteus 8.1 and OriginPro 2021.

#### 2.5.3 Dynamic Mechanical Analysis (DMA)

Thermo‐mechanical properties were assessed using a DMA 242 E Artemis from NETZCH in tensile mode. The samples were heated from −100 to 175 °C at 2 °C min^−1^ at a frequency of 1 Hz and a displacement amplitude of 10 µm (0.2%). Data were exploited using OriginPro 2021. The transition alpha temperature T_α_ values were taken at the maxima of tan (δ) peaks. The crosslink density was measured using the values of elastic storage modulus at 100 °C injected in Equation (8).

(8)

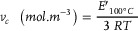




#### 2.5.4 Thermogravimetric Analysis (TGA)

TGA measurements were conducted on a TGA Q50 from TA Analysis. Samples were placed in 30 µL aluminum crucibles and heated from 25 to 580 °C at 20 °C min^−1^ under nitrogen flow (60 mL min^−1^). Data were exploited with Origin Pro 2021. T_2%_ was defined as the temperature at which the samples lost 2% of its initial mass.

#### 2.5.5 Gel Content

Gel contents (GC) were determined by exposing the materials to DMF for 24 h at 80 °C prior to washing (DMF) and vacuum drying overnight at 80 °C. Gel contents were calculated using Equation (9) and were *m_i_* stands for the initial mass of sample and *m_d_* for the mass of dried samples.

(9) $$Gel\;content\;=\left(1-\frac{m_{i}-m_{d}}{m_{i}}\right)\;\times 100$$

#### 2.5.6 Rheology Experiments

All the rheology experiments were conducted on an Anton‐Paar MCR 302 equipped with a H‐PTD200 Peltier.

The viscosity of NC514 and epoxy‐carbonate hybrid resins was measured from 0.1 to 100 rad s^−1^ at 25 °C using a 25 mm cone‐plate geometry. For the stress‐relaxation experiments, the rheometer was equipped with a plate‐plate sandblasted geometry (d = 8 mm). To ensure a proper contact between the plates, a 2 N axial force was applied for each experiment. The samples were subjected to a 2% (within the linear viscoelastic domain previously determined by strain sweep experiments) shear strain throughout the experiments. The relaxation modulus was monitored for 10^4^ or 10^5^ s at 150 °C for all samples. In addition, the evolution of the relaxation modulus of the selected hybrid (i.e., PHU‐47 with the best properties) was monitored overtime in isothermal mode at 130, 140, 150, and 160 °C. The characteristic relaxation time (τ) was obtained from stretched exponential fitting (Kohlrausch‐Williams‐Watts or KWW model) and employed to extract the activation energy (E_A_) from the Arrhenius plot (Figure S11B, Supporting Information).

## 3 Results and Discussion

The first section of this article is dedicated to the synthesis of partially carbonated cardanol‐based epoxy monomer (NC514 resins) and to the investigation of the dual reactivity of these resins with a primary diamine (MXDA, *m*‐xylene diamine). An expected advantage of combining ring‐opening of epoxy and cyclic carbonate by primary amine is to generate in situ amino‐alcohol moieties, thus avoiding the need of an exogeneous catalyst generally required for the aminolysis reaction.^[^ ^26^, ^30^ ^]^ The effect of carbonation on the curing kinetic, on the thermo‐mechanical, and the adhesion behavior of epoxy and epoxy‐PHU hybrids is discussed. Finally, insights on the stress‐relaxation behavior and reprocessability of catalyst‐free epoxy‐PHU hybrid materials are given.

### 3.1 Reactivity of Epoxy and Epoxy‐PHU Hybrids

Epoxy‐carbonate hybrid resins were prepared thought partial carbonation of cashew nutshell epoxy monomer (NC514) as illustrated in Figure 1A. The kinetic profile of carbonation determined by ^1^H NMR spectroscopy (80 °C, [NC514]_0_ = 0.5 g mL^−1^ of ethyl acetate, 5 wt.% of TBAB as catalyst) presented in Figure 1B allowed us to prepare four hybrid monomers containing respectively 33, 47, 80 and 88 mol% of cyclic carbonate. Recently, Cramail and co‐workers reported the partial carbonation kinetic of epoxidized soybean oil.^[^ ^27^ ^]^ In the case of epoxidized soybean oil (ESO), ≈25 h at 120 °C and 45 bars of initial CO_2_ pressure were required to reach 80% of conversion. Strikingly, even though the temperature and pressure conditions employed in our case were milder, 80% of conversion was achieved in only 2 h. Such discrepancy was attributed to the higher reactivity of glycidyl‐based epoxies on NC514 more prone to CO_2_, the latter known to be initiated by the ring‐opening of the oxirane.^[^ ^19^ ^]^

Four hybrid resins were synthesized, ranging from 33 to 88% of carbonation. Figure S2A (Supporting Information) presents the FTIR spectra of the prepared resins normalized to the aromatic peaks at 1580 cm^−1^. The normalized area of the carbonate peak (1750–1860 cm^−1^) was plotted with carbonate content obtained from ^1^H NMR quantification (Figure S2B, Supporting Information), thus demonstrating a rather good linear correlation (R^2^ = 0.996). Hence, after proper calibration, one could access the carbonate content of hybrid resins in a rapid and efficient manner.

5‐Membered cyclic carbonate monomers exhibit generally higher viscosity than their corresponding epoxides. The viscosity of NC514 (neat epoxide) and synthesized epoxy‐carbonate hybrids at 25 °C are presented in Figure S4 (Supporting Information). As expected, the viscosity of the studied resins progressively increased from 20 Pa.s for NC514 to 2 200 Pa.s with the increasing 5CC content. Controlling the viscosity, in this case over 2 orders of magnitude, of the initial formulation is of high interest for the preparation of thermoset materials and especially when designing adhesives (wettability).

**Figure 2:**
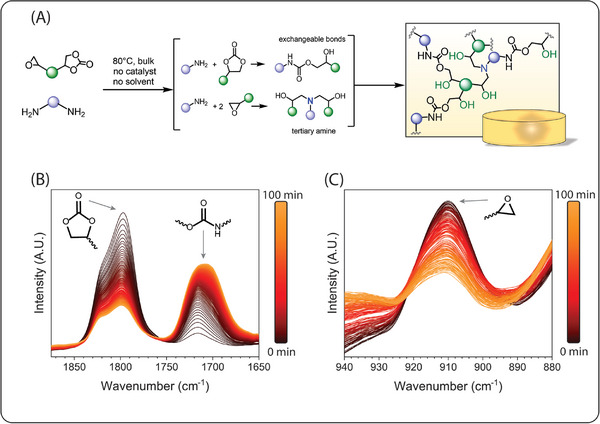
A) Polymerization of epoxy‐PHU hybrids, the reaction of primary amine with cyclic carbonate generates exchangeable hydroxyurethane bonds, and tertiary amines are generated by the double addition of primary amine on epoxide. B) ATR‐IR kinetic experiments of PHU‐88 and MXDA at 50 °C zoom on the carbonyl vibration region (1900–1650 cm^−1^) and (C) ATR‐IR kinetic experiments of NC514 and MXDA at 50 °C zoom on the epoxy region (940–880 cm^−1^).

Epoxy‐carbonate hybrid resins were then reacted with MXDA to produce hybrid materials. The synthesis route and an example of a possible structure is depicted in **Figure** **2**A.

To the best of our knowledge, the reactivity of partially carbonated intra‐chain epoxy monomers toward amines was assessed only once but not with glycidyl moities.^[^ ^27^ ^]^ To further strengthen the understanding of hybrid systems, the curing kinetic of MXDA with epoxidized cardanol (NC514), and partially carbonated NC514 (yielding PHU‐47 and PHU‐88) was investigated by FTIR in isothermal mode at 50 °C. Figure 2C respectively displays the magnifications of the carbonate/urethane and epoxy peaks of NC514 and PHU‐88. Spectra of PHU‐47 are also presented in Figure S4 (Supporting Information). The area of the carbonate peaks (1750–1860 cm^−1^) and the epoxy peaks (890–940 cm^−1^) were integrated for each spectrum. These data were injected in Equation (4) to obtain the conversion of epoxy and carbonate over time for NC514, PHU‐47, and PHU‐88 (Figure S5, Supporting Information). Kinetic plots (Figure S5, Supporting Information) highlighted a similar rate of conversion for the cyclic carbonate groups of both PHU‐47 and PHU‐88 throughout the polymerization but the ring‐opening of epoxy by primary amine occurred at a faster rate for PHU‐47 than for NC514 (neat epoxide). Such variations may originate from the higher weight percentage of amines employed in the polymerization of PHU‐47 than NC514 (≈1.4 time higher).

Helbling et al. highlighted a stepwise and selective process for the polymerization of epoxy‐PHU hybrids based on epoxidized soybean oil and 1,8‐octanediamine.^[^ ^27^ ^]^ After ≈10 min at 120 °C nearly 70% of the carbonate had already reacted while only 20% of the epoxy opened in the meantime. In the present system, both cyclic carbonates and epoxies react in a concomitant manner, a similar reactivity which was already described for molecular reactions (Figure S5, Supporting Information).^[^ ^35^ ^]^ This was attributed to the higher reactivity toward ring‐opening of glycidyl moieties of NC514 as compared to intra‐chain (disubstituted) epoxy groups of the modified vegetable oils. During kinetic experiments (Figure 2B), a progressive shift of the urethane's carbonyl vibration band from 1716 to 1708 cm^−1^ respectively to H‐bonded and free carbonyl vibration bands, occurred as the curing proceeds (Figure 2B).^[^ ^36^ ^]^ The FTIR spectra (zoom on the carbonyl region presented in Figure S6A, Supporting Information) of cured NC514 and epoxy‐PHU hybrids showed that the proportion of bonded carbonyl followed, as expected, the increase of initial carbonate content within the starting formulation. The ability of PHU to form a dense hydrogen‐bonded network was put forward to explain the better adhesion properties compared to neat epoxy or PU thermosets.^[^ ^9^, ^37^ ^]^

### 3.2 Thermo‐Mechanical Properties of Epoxy and Epoxy‐PHU Hybrids

**Figure 3:**
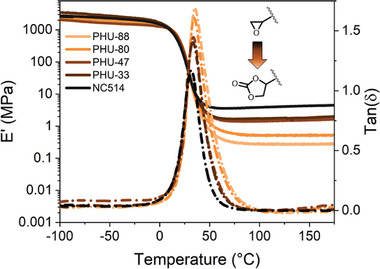
Elastic storage modulus (E’, MPa) and tan (***δ***) depend on the temperature of epoxy (NC514) and epoxy‐PHU hybrids. The numbers in the legend, next to “PHU‐XX” correspond to the percentage of carbonation of the starting formulations.

Thermo‐mechanical properties of epoxy (NC514) and epoxy‐PHU hybrids were determined by combining tensile tests, DMA, DSC, TGA and swelling tests, results are reported in **Table** **1**. Representative tensile curves are presented in Figure S8A (Supporting Information), TGA and DSC curves in Figure S8B,C (Supporting Information), respectively. Finally, **Figure** **3** showcases the DMA curves obtained from the materials manufactured from NC514 and partially carbonated NC514.

**Table 1:** Data extracted from the thermal and mechanical analysis of epoxy and epoxy‐PHU hybrids (tensile, DSC, DMA, TGA) as well as their gel content.

Sample	T_g_ ^a)^ [°C]	T_α _ ^b)^ [°C]	Stress at break [MPa]	Elongation at break [%]	E’_rubber_ ^c)^ [MPa]	GC [%]	T_2%_ [°C]
NC514	9	32	4.1 ± 0.6	200 ± 20	3.9	88.8 ± 2.8	324
PHU‐33	13	33	4.7 ± 0.3	260 ± 20	1.7	78.2 ± 1.8	291
PHU‐47	14	34	5.4 ± 1.3	310 ± 30	1.4	73.6 ± 0.4	279
PHU‐80	14	34	3.2 ± 0.4	500 ± 40	0.5	51.3 ± 1.0	273
PHU‐88	19	35	2.0 ± 0.3	420 ± 30	0.3	44.6 ± 0.3	266

^a)^ Determined on the second ramp of DSC analysis;

^b)^ Determined at the max(tan δ) of DMA analysis;

^c)^ Elastic storage modulus at 100 °C.

DSC and DMA analysis respectively show that the T_g_ of the synthesized thermosets were comprised between 9 and 19 °C and their T_α_ between 32 and 35 °C. Such small variations of T_g_ and T_α_ with the degree of carbonation suggest, at first glance, a negligible impact of the partial carbonation (introduction of hydroxy urethane moieties) on the thermomechanical of the final materials. Yet, the effect of carbonation was more pronounced on the mechanical properties. Tensile tests showed that hybrid materials prepared from monomer containing 33 and 47 mol% of carbonate increases both the stress and strain at break, thus improving the properties of neat epoxy resins. However, a further increase in carbonate content up to 80% lead to a drastic drop of the mechanical properties. In addition, the decrease of the elastic storage modulus (E’_rubber_ at 150 °C) from 3.9 to 0.3 MPa and gel content (from 88.8% to 44.6%), measured respectively by DMA and gel content determination in DMF, also confirmed the impact of carbonation on the thermomechanical properties. This phenomenon is readily explained by the evolution of the chemical network topology as the carbonate content is rising. As a matter of facts, primary amines are able to ring‐open two epoxy groups while they react just once with a cyclic carbonate function. Thus, increasing the initial feed ratio [carbonate]_0_/[epoxy]_0_ will inevitably lead to a thermoset with reduced crosslink density. This assumption was confirmed by plotting. This assumption was confirmed using the elastic storage modulus at 100 °C determined by DMA analysis and further injected these values in Equation (8). As expected and depicted in Figure S9 (Supporting Information), the evolution of crosslink density with the increasing carbonate content decreases in a linear manner.

Lambeth et al. formulated and characterized epoxy‐PHU hybrids from the copolymerization of trimethylolpropane triglycidyl ether and its fully carbonated analogue. In their study, the tan (δ) peak of epoxy‐PHU hybrids could spread over a range of 125 °C with significant shouldering, showcasing the rather high heterogeneity of the chemical networks (epoxy monomer viscosity is four orders of magnitude lower than the fully carbonated derivative, see Figure S7, Supporting Information). In Figure 3 and better appreciated in its magnified version of Figure S9 (Supporting Information), the tan (δ) peak of epoxy‐PHU hybrids with carbonation percentage between 33 and 88% spreads over 100 °C. Yet, the tan (δ) peaks of PHU‐33 to 88 is much more gaussian than previous works. Hence, partially carbonating multifunctional epoxy monomers, as depicted in Figure 2A, allows to overcome the heterogeneity encountered when formulating 100% epoxy and 100% carbonate monomers together.

**Figure 4:**
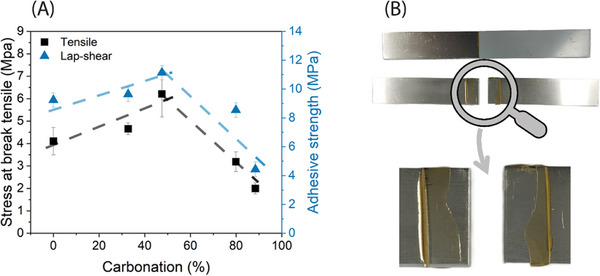
A) Stress at break (black squares) and adhesive strength (blue triangles) of epoxy and epoxy‐PHU hybrids. B) Picture of lap‐shear test before and after failure (adhesive/cohesive failure).

Lap‐shear and tensile test results, summarized in **Figure** **4**A, present the adhesive strength (on aluminum substrate) and the tensile stress at break of NC514‐based epoxy‐PHU hybrids. For the lap‐shear test, the adhesive strength progressively increases to reach a maximum of 12 MPa when increasing the carbonation ratio from 0 to 47%. Increasing this ratio to higher values yield to a significant decrease of the adhesion strength from 12 to 4 MPa (at 88% carbonated epoxy functions). Previously reported adhesion strength on aluminum substrates were comprised between 7 and 27 MPa for hybrid systems, higher values generally obtained from highly crosslinked and/or high T_g_ materials (e.g., trimethylolpropane triglycidyl ether or bisphenol A diglycidyl ether derivatives).^[^ ^9^, ^23^ ^]^ Regardless of the formulation, the mechanism of bond failure was cohesive/adhesive as depicted in Figure 4B. The tensile stress at break of bulk materials follows the same trend as measured in lap‐shear test with an optimum at roughly 50 mol% of carbonation and a significant drop at higher ratio. As mentioned, two competitive factors may explain the variations in mechanical properties and adhesive strength. The formation of a dense hydrogen bonded network by increasing the proportion of hydroxy‐urethane moieties is generally put forward to explain the improvement of mechanical and adhesion properties. On the other hand, increasing the concentration of 5CC moieties lead to a lower crosslink density (vide supra) and consequently reduced mechanical properties. The adhesion mechanism is quite complex and depends on both molecular interactions (nature of the chemical bonds and/or weak interactions between the adherend and the adhesive) and macroscopic phenomenon through the penetration of adhesives into pores, cavities, and other surface irregularities on the surface of the substrate (wettability). Apart from being excellent hydrogen bonds promoter, hydroxy‐urethane groups are also known as exchangeable covalent bonds and can be employed to design covalent adaptable networks or CANs.^[^ ^30^, ^38^, ^39^ ^]^ The peculiar rheological behavior of CANs (thermosets able to flow) has already shown to enhance the adhesion properties of epoxy resins (with exchangeable disulfide bonds) by avoiding the formation of trapped voids and the accumulation of stress.^[^ ^34^ ^]^ This behavior may also explain the superior adhesion properties of hybrid PHU as compared to neat epoxy thermosets.

### 3.3 Vitrimer Behavior and Reprocessability of Epoxy and Epoxy‐PHU Hybrids

**Figure 5:**
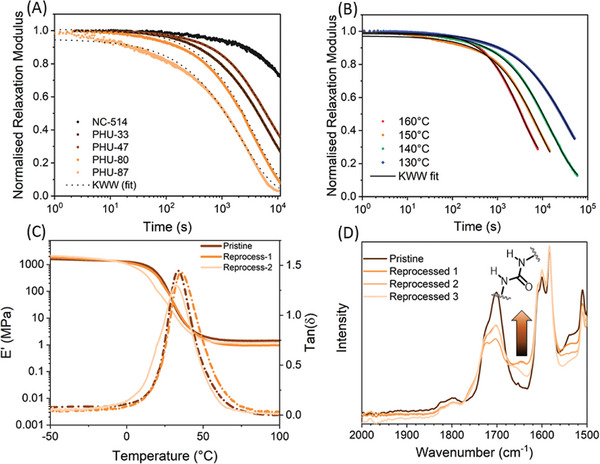
A) Stress‐relaxation plots (and KWW fitted curves) for N514 and epoxy‐PHU hybrids at 150 °C. B) Stress‐relaxation plots for PHU‐47 from 130 to 160 °C (C) DMA analysis of PHU‐47 pristine and after two reprocessing cycles. D) Magnified FTIR spectra (2000–1500 cm^−1^) of pristine and reprocessed PHU‐47 (up to three times).

To study the influence of partial carbonation on the rheological properties of hybrid PHU, stress relaxation experiments were first performed on all sample at 150 °C. As expected, increasing the concentration of exchangeable groups led to a faster relaxation with characteristic relaxation times (τ_KWW_) decreasing from 8 500 seconds for PHU containing 47 mol% of 5CC to 2200 s for the PHU containing 88 mol% of cyclic carbonates. The non‐normalized data presented in Figure S11A (Supporting Information) also demonstrated the significant drop of material properties with an initial relaxation shear modulus (G_0_) ranging from 0.5 MPa to 30 kPa while increasing the carbonate content, also consistent with the evolution of elastic modulus recorded in DMA (Figure 3). Interestingly, materials prepared from 33 and 47 mol% carbonated NC514 still exhibited good mechanical properties (shear and elastic modulus at 150 °C) close to the neat epoxy resin. The activation energy E_A_ = 124 kJ mol^−1^ measured from the Arrhenius plot presented in **Figure** **5**B (and Figure S11B, Supporting Information) for PHU‐47 is similar of values reported in the literature for catalyzed ^[^ ^28^ ^]^ (dibutyl tin dilaureate) and internally catalyzed transcarbamoylation^[^ ^32^ ^]^ (the exchange of hydroxyl group and carbamate functions). This result suggests that the presence of tertiary amine, resulting from the ring‐opening of two oxirane moieties by a primary amine, can efficiently promote transcarbamoylation. Alternatively, the high concentration of hydroxyl groups also resulting from the epoxy ring‐opening could also explain the improved relaxation properties. To go further, reprocessing experiments on the selected PHU‐47 (materials exhibiting the best compromise between mechanical, adhesion, and relaxation properties) were performed by grinding the samples into small pieces and hot‐pressed at 150 °C under 3 tons for 6 h (Figure S12, Supporting Information). DMA, ATR‐IR, and tensile tests were performed on reprocessed samples and confirmed the good reprocessability of the PHU‐47 sample. However, when performing multiple reprocessing cycles, we observed that the recovery of mechanical properties decreased after the second cycle. For instance, the three‐time reprocessed PHU‐47 was solely characterized by FTIR due to macroscopic defects preventing any accurate thermomechanical analysis. After two reprocessing cycles, DMA showed identical T_α_ (from 32 °C < T_α_ < 37 °C) and a slight decrease of the elastic modulus at 100 °C (E’ (pristine) = 1,43 MPa and E’ (2^nd^ repro) = 0,96 MPa, Figure 5C; Figure S13, Supporting Information). These results combined with the increased intensity of a vibration band associated to urea bonds at 1650 cm^−1^ suggests the occurrence of side reactions. Our group previously demonstrated that the introduction of a catalyst into a PHU matrix can influence both the mechanism and the efficiency of the exchange reaction.^[^ ^30^ ^]^ Tertiary amine such as DMAP (4‐(Dimethylamino)pyridine) or strong organic bases such as DBU (1,8‐Diazabicyclo[5.4.0]undec‐7‐ene) and TBD (1,5,7‐triazabicyclo[4.4.0]dec‐5‐ene) were found to preferentially promote the formation of urea via dissociative transcarbamoylation at high temperatures, that is, via the retro‐formation of cyclic carbonate and primary amine. Nevertheless, the approach based on partial carbonation exhibits better reprocessability than those prepared with an exogenous catalyst.^[^ ^30^ ^]^

## 4 Conclusion

The partial carbonation of epoxy monomers appears as a promising and simple pathway to tune the thermomechanical and viscoelastic properties of PHU thermoset formulations. This monomer design allows to control the viscosity of the starting formulation (over several order of magnitude) thus improving the homogeneity of the final material compare to a simple mixture of neat epoxides and cyclic carbonates. The presence of up to 47 mol% of hydroxy‐urethane moieties, able to create a dense hydrogen network, enhanced the maximum adhesion strength up to 20% as compared to neat epoxide while keeping similar thermo‐mechanical properties (T_g_, tensile properties, shear, and elastic modulus). The introduction of exchangeable groups within the epoxy matrix also brings additional properties such as reprocessability thanks to internally catalyzed transcarbamoylation.

## Conflict of Interest

The authors declare no conflict of interest.

## Supporting information

Supporting Information

## Data Availability

The data that support the findings of this study are available in the supplementary material of this article.
