# Supplementary material for: Controlling Hybrid Polyhydroxyurethane Adhesive and Rheological Properties by Partial Carbonation of Biobased Epoxy Monomer
Source: Macromol Rapid Commun. 2024 Jul 29;45(23):2400542. doi: 10.1002/marc.202400542 (PMC11628358; doi:10.1002/marc.202400542)
Supplement: Supplementary file 1 — Supporting Information [file MARC-45-2400542-s001.docx]

*Supplementary Information*

*Controlling Hybrid Polyhydroxyurethane Adhesive and Rheological Properties by Partial Carbonation of Biobased Epoxy Monomer*

Pierre Dellière,^a^ Dorian Laborie, ^a^ Sylvain Caillol, ^a^* Camille Bakkali-Hassani ^a^*

**Table S1.** Carbonation time, carbonate content and epoxy content of NC514 and partially carbonated NC514 determined by ^1^H NMR titration.

| **% of carbonation** | **Carbonation time (min)** | **Carbonate content (mmol.g^-1^)** | **Epoxy content (mmol.g^-1^)** |
| --- | --- | --- | --- |
| 0 (NC514) | 0 | 0.00 ± 0.00 | 2.07 ± 0.07 |
| 33 | 25 | 0.67 ± 0.06 | 1.32 ± 0.07 |
| 47 | 48 | 0.98 ± 0.07 | 1.24 ± 0.08 |
| 80 | 104 | 1.65 ± 0.19 | 0.41 ± 0.03 |
| 88 | 240 | 1.82 ± 0.13 | 0.20 ± 0.08 |


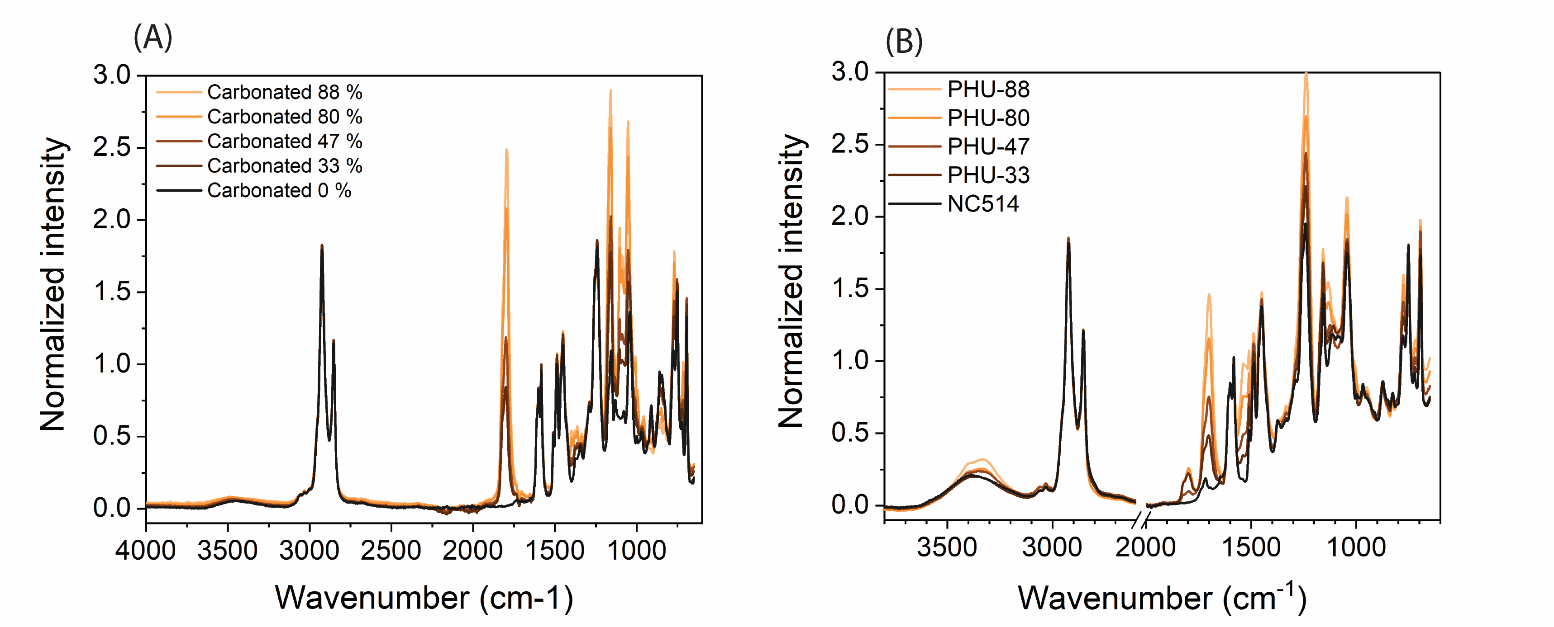


**Figure S1.** (A) FTIR spectra of NC514 and partially carbonated NC514 resins. (B) FTIR spectra of epoxy and epoxy-PHU hybrids. The numbers in the legend correspond to the percentage of carbonation.

**Table S2.** Masses of NC514, partially carbonated NC514 and MXDA for each PHU hybrid

| **Sample** | **Mass of NC514 or partially carbonated NC514 (g)** | **Mass of MXDA (g)** |
| --- | --- | --- |
| NC514 | 12.88 | 0.91 |
| PHU-33 | 12.87 | 1.17 |
| PHU-47 | 13.65 | 1.33 |
| PHU-80 | 13.42 | 1.69 |
| PHU-88 | 13.12 | 1.72 |


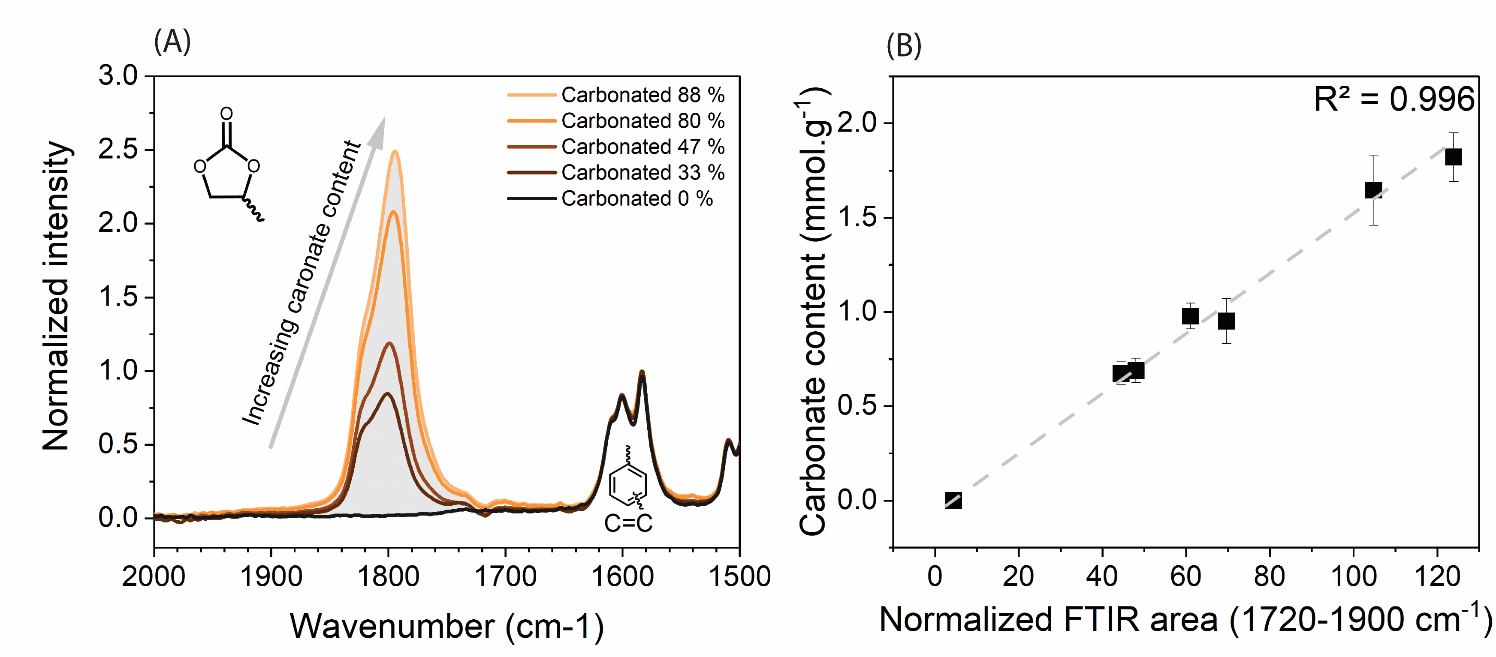


**Figure S2.** (A) Normalized and magnified FTIR spectra of NC514 and partially carbonated NC514. (B) Linear relationship between the normalized FTIR carbonate area (1710-1880 cm^-1^) and the carbonate content.





**Figure S3.** Viscosity of NC514 and partially carbonated NC514 from 0.1 to 100 s^-1^.


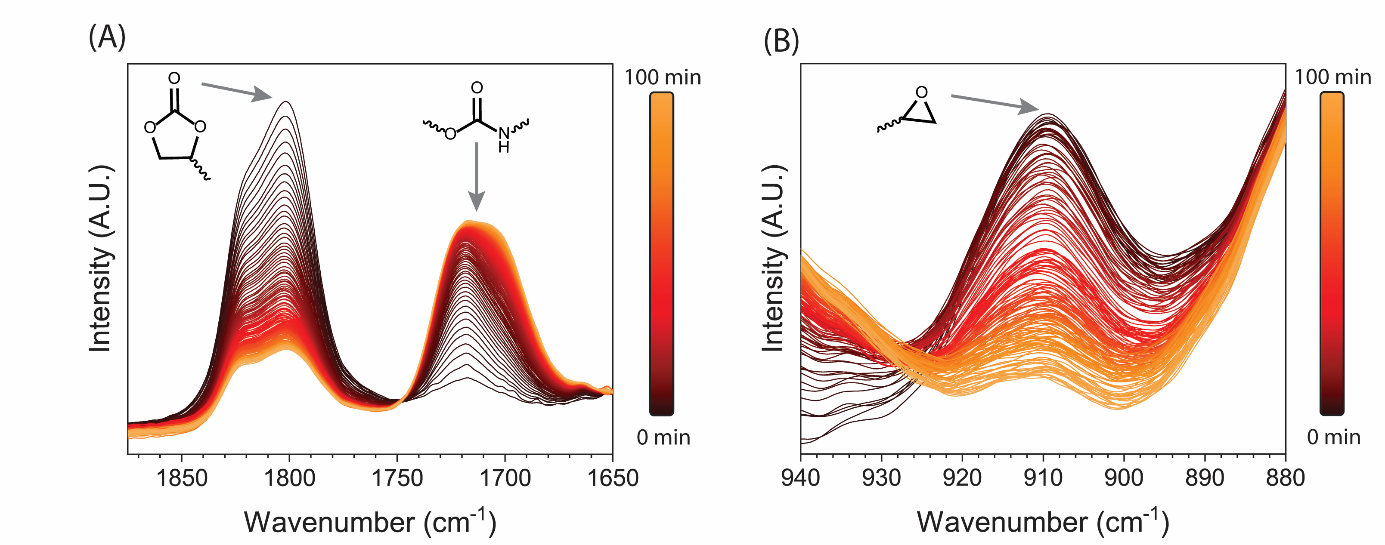


**Figure S4.** FTIR spectra of PHU-47over the course of the polymerization at 50 °C magnified on the carbonate/urethane area (A) and on the epoxy area (B).


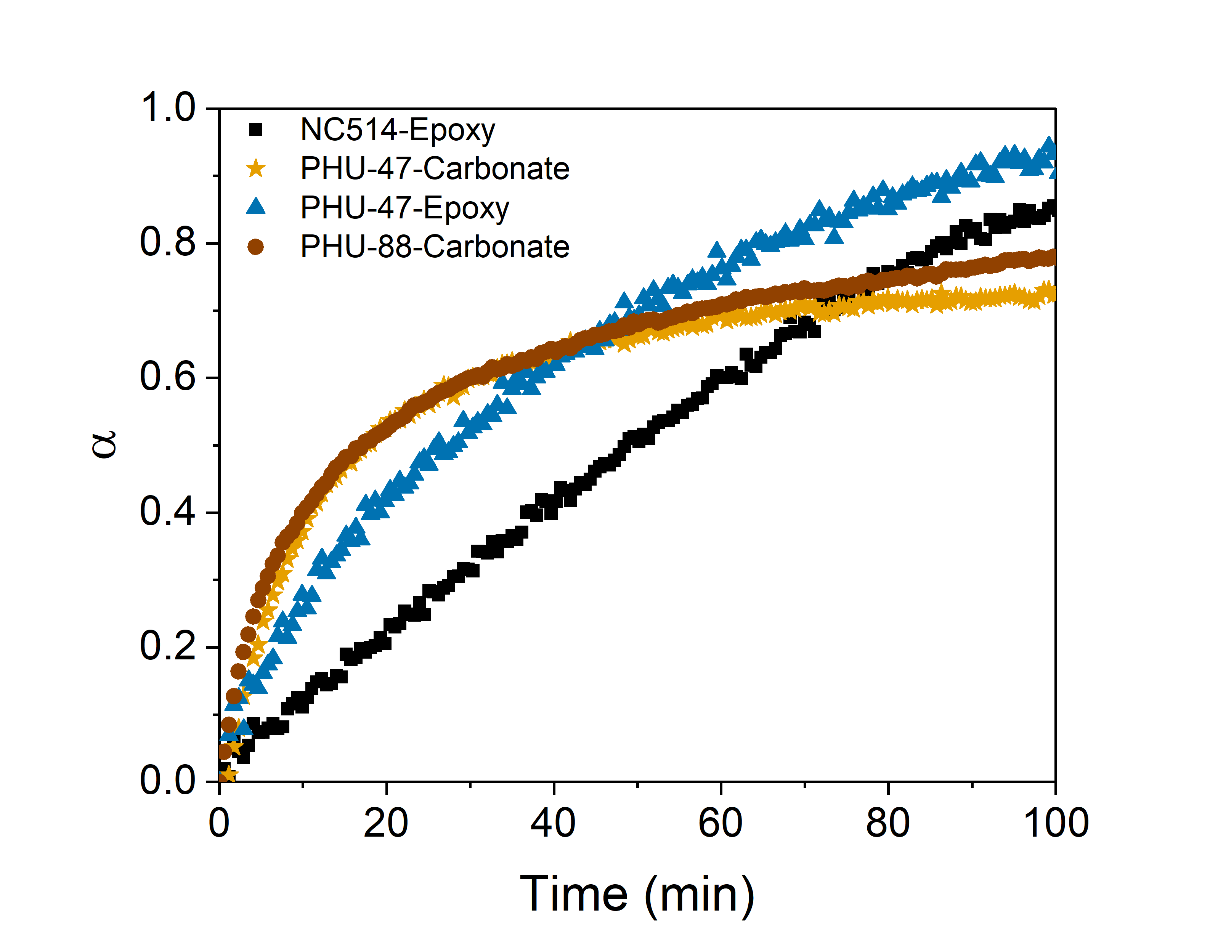


**Figure S5.** Conversion of epoxy and cyclic carbonate group as function of time measured by ATR-IR at 50°C for NC514, PHU-47 and PHU-88.


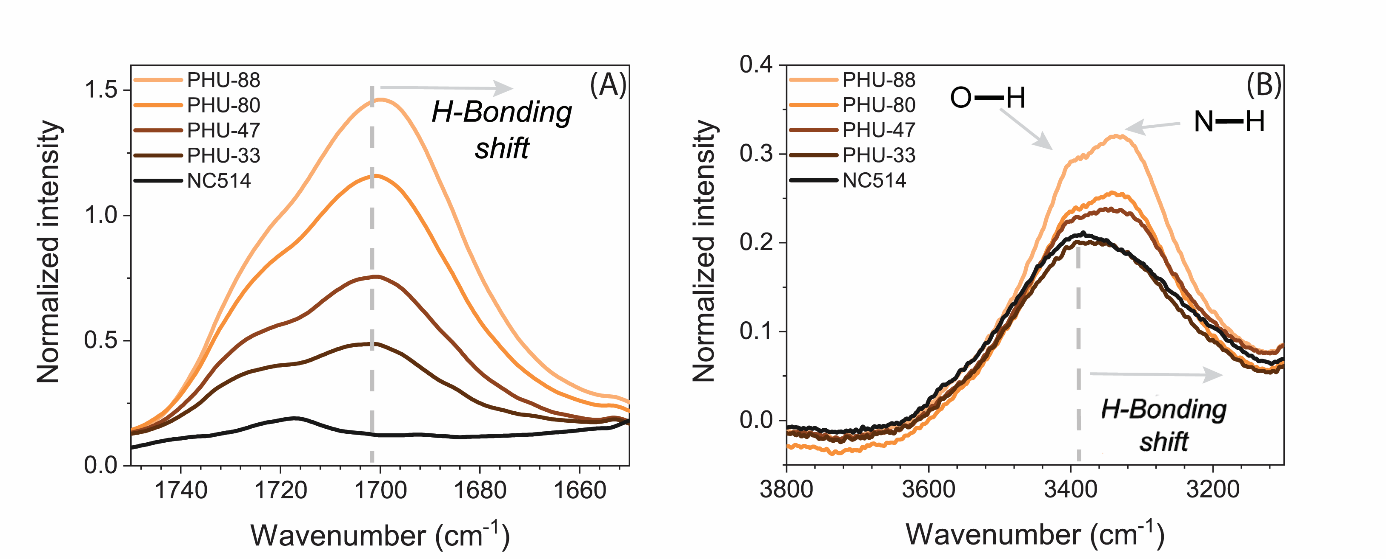


**Figure S6.** Magnified FTIR spectra of cured NC514 and epoxy-PHU hybrids on the urethane area (A) and the OH/NH area (B).


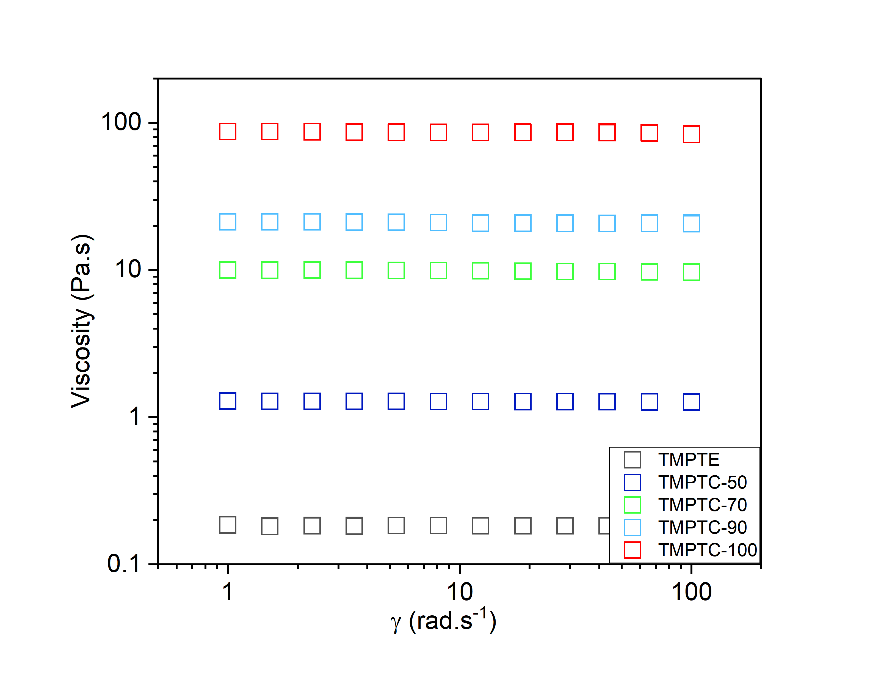


**Figure S7.** Viscosity of trimethylolpropane triglycidyl ether as a function of mol% carbonation.


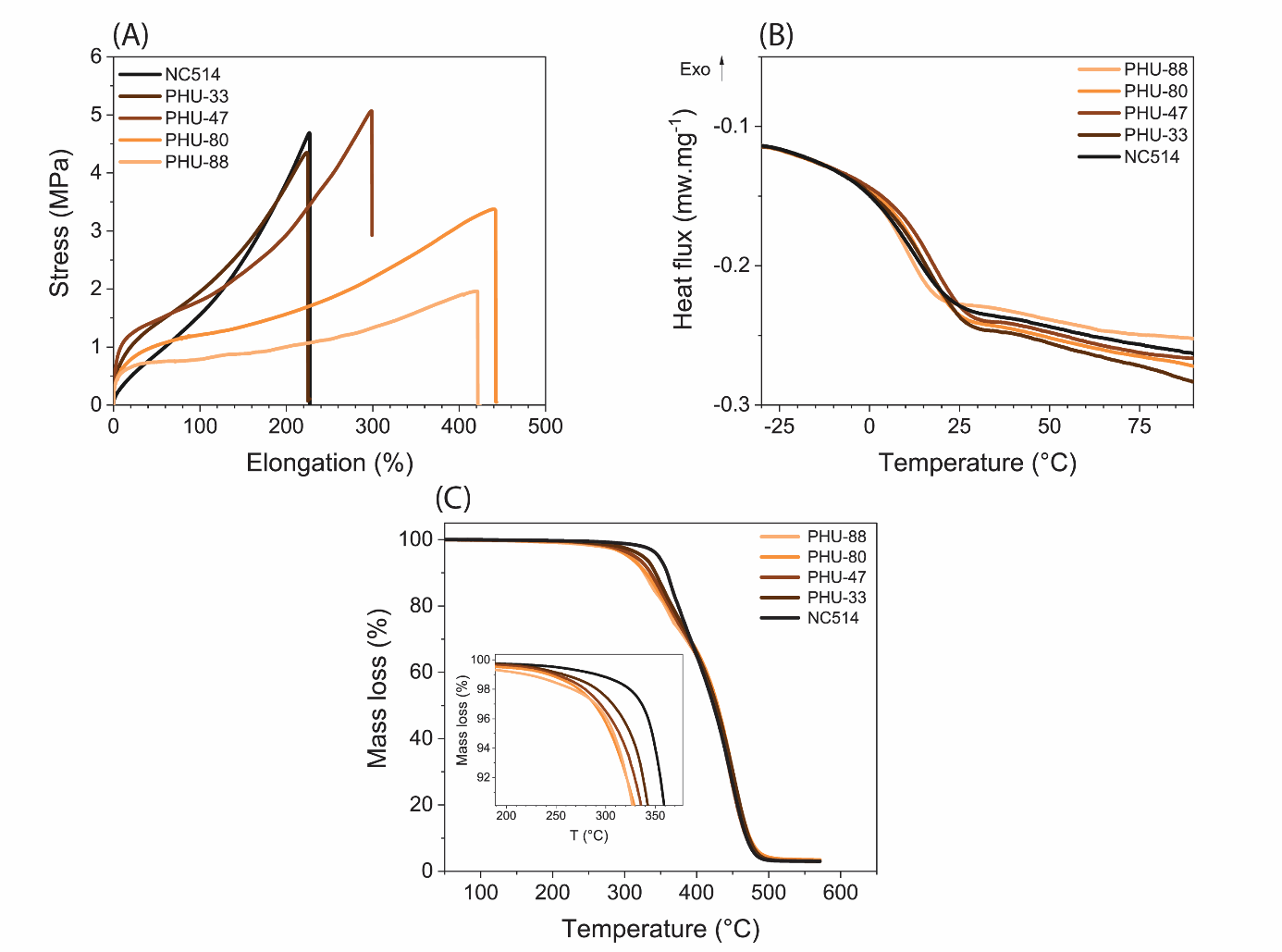


**Figure S8.** Representative tensile (A), DSC (B) and TGA (C) curves of cured NC514 and epoxy-PHU hybrids


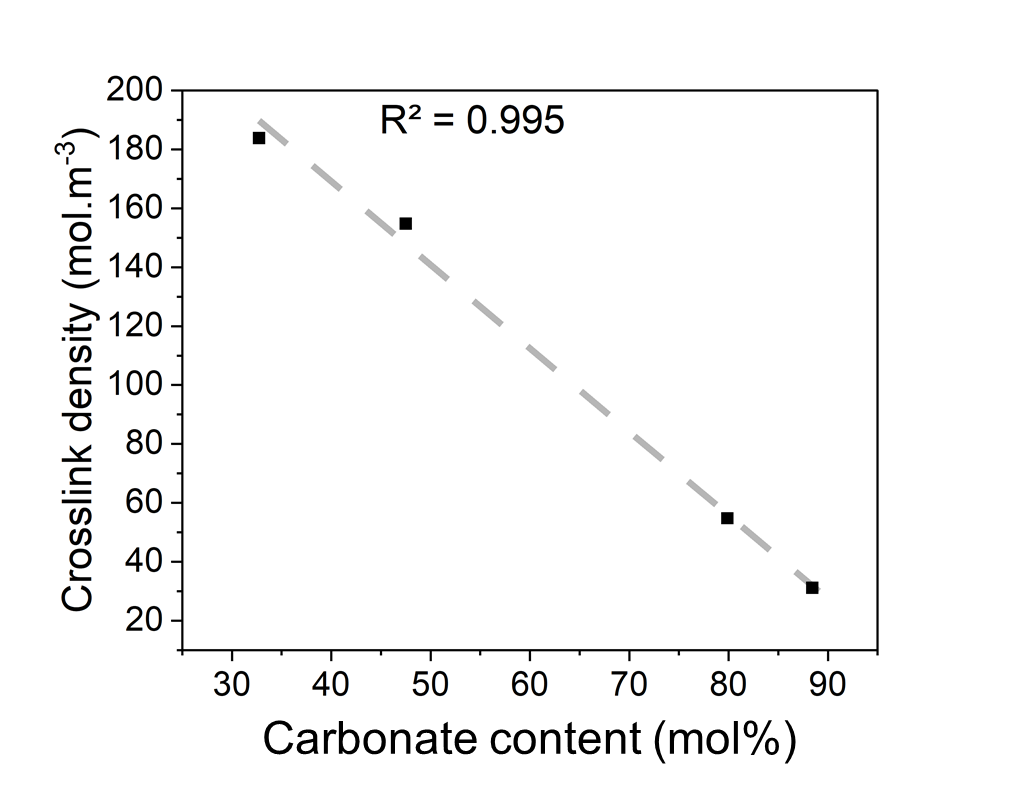


**Figure S9.** Evolution of the crosslink density as a function of carbonate content, calculated from the elastic storage modulus at 100°C and Equation 8.





**Figure S10.** Magnified tan ($\delta$) curve of cured NC514 and epoxy-PHU hybrids


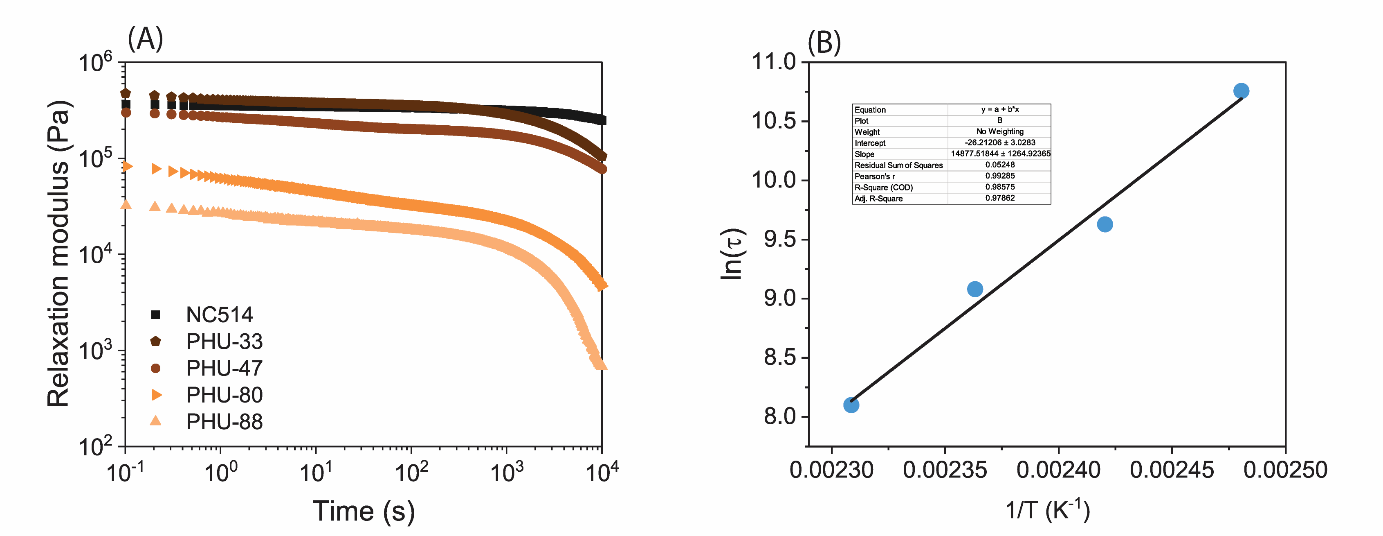


**Figure S11.** (A) Non-normalized stress relaxation curves of materials prepared from partially carbonated NC-514 and neat NC-514 at 150°C (B) Arrhenius plot of PHU-47 (extracted from isothermal stress relaxation experiments 130°C, 140°C, 150°C and 160°C).


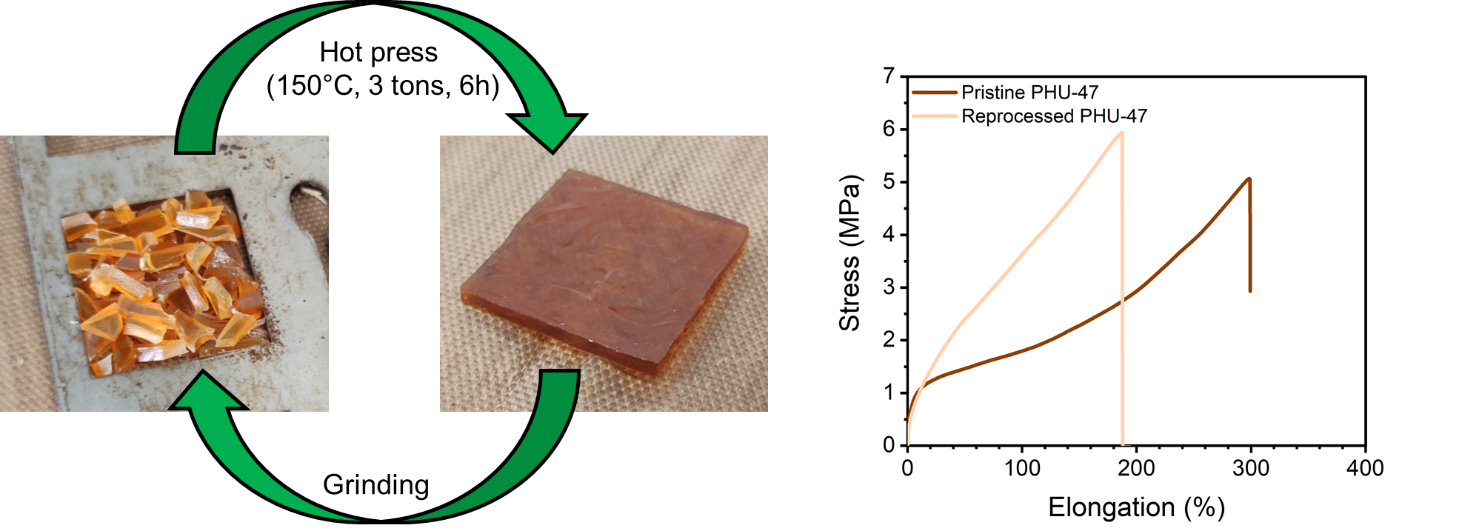


**Figure S12.** (A) Pictures and tensile tests before and after reprocessing for PHU-47 (conditions: 6h, 150°C, 3 tons).


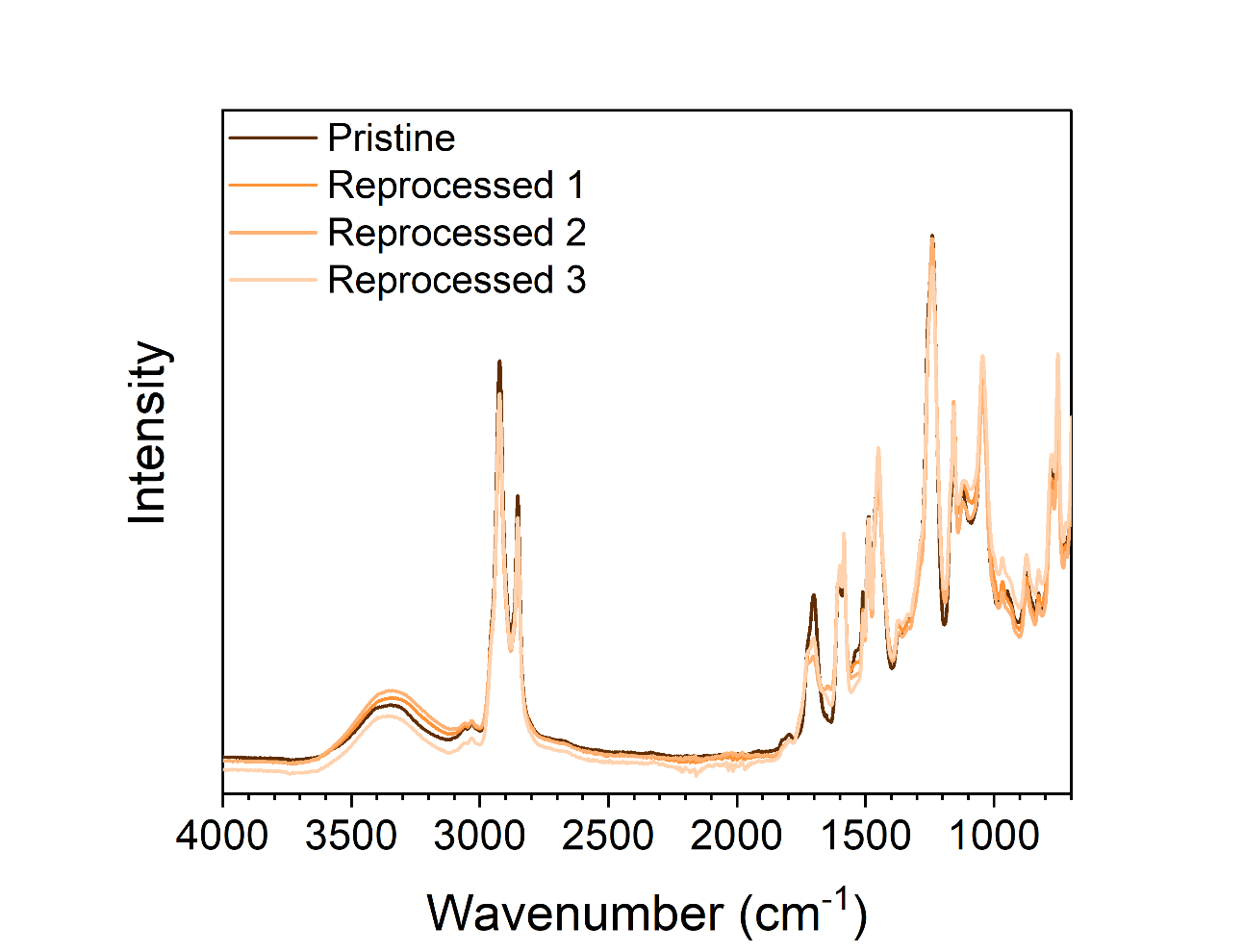


**Figure S13.** FTIR spectra of PHU-47 pristine and after each reprocess cycles
